# Journal of clinical monitoring and computing end of year summary 2018: hemodynamic monitoring and management

**DOI:** 10.1007/s10877-019-00297-w

**Published:** 2019-03-07

**Authors:** Bernd Saugel, Moritz Flick, Karim Bendjelid, Lester A. H. Critchley, Simon T. Vistisen, Thomas W. L. Scheeren

**Affiliations:** 1 Department of Anesthesiology, Centre of Anesthesiology and Intensive Care Medicine, University Medical Centre Hamburg- Eppendorf, Martinistrasse 52, 20246 Hamburg, Germany; 2 Department of Anesthesiology and Intensive Care, Geneva University Hospitals, Geneva, Switzerland; 3 Department of Anesthesia and Intensive Care, The Chinese University of Hong Kong, Shantin, N.T., Hong Kong; 4 The Belford Hospital, Fort William, The Highlands, Scotland UK; 5 Department of Clinical Medicine, Aarhus University, Aarhus, Denmark; 6 Department of Anesthesiology, University Medical Center Groningen, University of Groningen, Groningen, The Netherlands

**Keywords:** Hemodynamic monitoring, Blood pressure, Blood flow, Fluid responsiveness, Cardiovascular physiology, Cardiac output, Closed loop, Hypotension, Perioperative outcome

## Abstract

Hemodynamic management is a mainstay of patient care in the operating room and intensive care unit (ICU). In order to optimize patient treatment, researchers investigate monitoring technologies, cardiovascular (patho-) physiology, and hemodynamic treatment strategies. The Journal of Clinical Monitoring and Computing (JCMC) is a well-established and recognized platform for publishing research in this field. In this review, we highlight recent advancements and summarize selected papers published in the JCMC in 2018 related to hemodynamic monitoring and management.

## Introduction

Hemodynamic management is a mainstay of patient care in the operating room and intensive care unit (ICU). In order to optimize patient treatment, researchers investigate monitoring technologies, cardiovascular (patho-) physiology, and hemodynamic treatment strategies. The Journal of Clinical Monitoring and Computing (JCMC) is a well-established and recognized platform for publishing research in this field. In this review, we highlight recent advancements and summarize selected papers published in the JCMC during the last year related to hemodynamic monitoring and management.

## Blood pressure monitoring

Blood pressure (BP) measurement is essential in many fields of health care, especially during surgery and in an intensive care setting. As an alternative to invasive BP measurements, several safe and easy to use non-invasive technologies have been proposed for continuous BP monitoring. In order to become reliable alternatives to invasive or intermittent oscillometric upper-arm cuff measurements these devices need to overcome limitations [1, 2]. In 2018, the JCMC published several interesting articles on technologies for non-invasive BP monitoring.

In a randomized controlled trial, Juri and co-workers [3] compared the agreement between intermittent oscillometric upper arm cuff measurements and continuous non-invasive measurement using the vascular unloading technique (finger cuff method) with the ClearSight system (Edwards Lifesciences, Irvine, CA, USA; formerly Nexfin, BMEYE, Amsterdam, The Netherlands) during knee arthroplasty. Another objective of the study was to investigate if continuous BP monitoring can reduce the incidence of hypo- or hypertension during general anesthesia. A total of 40 patients were included in the final analyses and five patients were excluded due to arrhythmia or impaired cardiac function. Overall, the ClearSight system showed a mean of the differences with a standard deviation (SD) of − 3.9 ± 10.3 mmHg, however the respective results for systolic, diastolic and mean BP were not reported. The agreement between the methods was better during the intraoperative phase (− 1.1 ± 8.1 mmHg) compared to the induction phase (− 8.7 ± 14.4 mmHg) and the end of anesthesia (− 9.9 ± 9.4 mmHg). Additional analyses included a four-quadrant plot with a concordance rate of 95.1% and a polar plot with an angular bias of − 9.3° ± 21.4° and concordance rate of 79.2%. In line with previous studies, continuous BP monitoring led to better hemodynamic stability (defined as a systolic BP between 80 and 110% of the baseline value) and fewer incidences of hypotension during induction and maintenance of anesthesia. A limitation of the study was the lack of an arterial catheter for continuous invasive BP measurements as a reference method. Based on their findings, the authors concluded that the ClearSight system may help to improve patient care during general anesthesia, when invasive continuous BP monitoring is not possible or unjustifiable.

BP measurement in patients with atrial fibrillation is challenging due to beat-to-beat BP variation. The direct measurement of BP with an arterial catheter is the commonly preferred method, but—due to its invasiveness—is not warranted in most patients and practically not feasible in clinical settings outside the operating room or ICU. Berkelmans et al. [4] proposed that the finger cuff/volume clamp method might be a solution for this problem. The authors performed a prospective method comparison study to compare BP measurements from an invasive arterial catheter and from the non-invasive volume clamp monitor Nexfin (as stated above this is now called ClearSight). The authors included 41 patients in medium care units or ICUs, 31 patients with atrial fibrillation and 10 patients with sinus rhythm. Despite the challenging setting, Bland–Altman analysis showed a low mean of the differences and SD in patients with atrial fibrillation for mean BP (0 ± 8 mmHg) and diastolic BP (1 ± 7 mmHg), but not for systolic BP (− 4 ± 12 mmHg). The absolute beat-to-beat systolic BP differences (1.5 mmHg, interquartile range 0.5–3.8 mmHg) were small between the volume clamp method and the direct arterial measurement method indicating that the Nexfin system can detect BP variability due to atrial fibrillation. Overall, the results in patients with sinus rhythm were similar to those in patients with atrial fibrillation. A limitation of the study was that the Nexfin device showed a high mean of the differences and wide limits of agreement in one-third of all patients in both groups, possibly due to edema or inadequate peripheral perfusion. Nevertheless, the results are promising and the authors concluded that their findings should encourage future studies investigating the impact of atrial fibrillation on the measurement performance of BP monitoring methods.

Lakhal et al. [5] also contributed an interesting study on BP monitoring in arrhythmic patients. The authors simultaneously measured invasive arterial BP and standard intermittent non-invasive oscillometric BP in a cohort of 216 ICU patients, 127 with sinus rhythm and 89 with arrhythmia. Additionally, the authors performed an intervention (e.g. volume expansion, passive leg raising, initiation/change in dosage of vasopressors or inotropic medications, or combination of these interventions) in patients with acute circulatory failure. The findings showed a relatively high mean of the differences (± SD) between intermittent oscillometry- and arterial catheter-derived BP measurements in both patients with arrhythmia (mean BP 9.1 ± 9.5 mmHg) and sinus rhythm (mean BP 9.1 ± 7.3 mmHg). Furthermore, the differences in systolic, diastolic, and mean BP were comparable between the groups with a tendency of larger differences in patients with sinus rhythm. Using an average of three measurements significantly reduced the SD of the mean of the differences in patients with arrhythmia (mean BP 9.1 ± 9.5 mmHg vs. 9.1 ± 7.4 mmHg, p < 0.01), but not in patients with regular rhythm. The area under the receiver operating characteristics curve (AUC_ROC_) for the detection of hypo- and hypertensive patients (AUC_ROC_: 0.88–0.92), as well as cardiovascular intervention responders (AUC_ROC_: 0.81 vs. 0.83) were similar in both groups. The authors concluded that the performance of the oscillometric BP device was not worse in patients with arrhythmia compared to patients with regular heart rhythm. The observed mean of the differences between oscillometric and invasive BP measurements indicates that these two clinically established BP monitoring methods are not interchangeable.

Continuous non-invasive BP measurement using radial artery applanation tonometry is another innovative concept and has shown promising results in the past [6, 7]. For the first time, Harju et al. [8] compared BP values obtained with the modified arterial tonometry sensor BPro (HealthSTATS International, Singapore) and invasively assessed BP values in a postoperative setting. Overall, the recordings in 28 patients showed large differences between the methods. Bland–Altman plots indicated poor agreement for systolic (mean of the differences ± SD, 19.8 ± 16.7 mmHg) and mean BP (11.2 ± 11.1 mmHg). However, in accordance with a previous study [9], the results indicate that radial artery tonometry estimates diastolic pressures more accurately and precisely (4.8 ± 7.7 mmHg). The four-quadrant plot used to determine the trending ability of the test method showed a low concordance with the reference method (48.7%). A further limitation of the modified BPro sensor remains a high rate of failed measurements (21.6%), which further increased in this cohort due to movement of the patient or sensor.

In the October issue, Greiwe et al. [10] evaluated the performance of automated radial artery applanation tonometry using the T-Line 400 (Tensys Medical, San Diego, CA, USA) in awake or anesthetized patients in a cardiological ICU. The authors conducted a total of 27,900 measurements in 31 patients with severe cardiac comorbidities, including highly impaired left ventricular function, atrial fibrillation, and severe aortic valve stenosis (< 1.0 cm^2^). The results showed substantial disagreement between BP assessed using radial artery applanation tonometry and invasively measured BP. The findings in this cohort are in contrast to previous studies in general ICU patients which found better agreement between BP readings obtained using automated radial artery applanation tonometry and invasive BP measurements [6, 11, 12]. However, the authors pointed out the fact that BP data were not averaged as aggregation of data can lead to underestimation of measurement differences. Additionally, the authors explained the poor measurement performance with the overall impaired hemodynamics in this cohort due to cardiologic morbidities. Three patients were excluded due to excessive movement; in the remaining patients, minor limb movement did not affect the results, which were comparable between awake and anesthetized patients. On a side note, the results showed that automated radial artery applanation tonometry, in this study, tended to underestimate the invasively measured BP during hypertensive episodes, while BP was overestimated in hypotensive periods. Therefore, the authors concluded that radial artery applanation tonometry is not reliable for BP monitoring in patients with severe cardiac morbidities.

A novel device to continuously record non-invasive BP was introduced by Földi et al. [13] in the August issue. The OptoForce device (OnRobot A/S, Odense, Denmark) is placed over the radial artery and uses a three-axial force sensor based on infrared light reflection to detect the BP signal. In their proof of principle study, the authors compared the innovative OptoForce to a Millar tonometer (Millar Instruments, Houston, TX, USA), a handheld non-invasive probe which can obtain BP waveforms, in 30 healthy young volunteers. Data were recorded after calibrating both, the novel OptoForce and the arterial tonometry device, to the same upper arm oscillometric BP signal. Bland–Altman analysis showed a low mean of the differences with small SD for systolic (0.35 ± 1.75 mmHg), diastolic (0.02 ± 0.19 mmHg), and mean (2.88 ± 2.42 mmHg) BP. With these promising results, the authors suggested future studies to compare the novel device to invasive arterial BP. However, the findings have to be interpreted carefully as the recorded data are from a highly selected cohort of patients and were averaged before analysis.

## Blood flow monitoring

In 2018, various papers on blood flow monitoring were published in the JCMC. There was only one paper, however, that looked directly at the performance of currently available cardiac output (CO) monitors in the clinical setting. One study evaluated a new prototype method, while other papers investigated external factors including obesity, auto-calibration, and choice of operator. Finally, one paper looked at using a CO monitor in high-fidelity patient simulations.

Lamia et al. [14] performed a cross comparison of trending accuracies of four currently marketed CO monitors using pulse contour analysis (LiDCOplus, LiDCO Ltd., London, UK; FloTrac, Edwards Lifesciences; PiCCOplus, Pulsion Medical Systems SE, Feldkirchen, Germany) and bioreactance (NICOM, Cheetah Medical, Newton Center, MA, USA) to estimate CO. Pulmonary artery catheter thermodilution (Edwards Lifesciences) was also included, with a pooled reference CO averaged from all five techniques. Data were collected from 21 post cardiac surgery patients during their first 2 h of ICU admission. In addition to Bland–Altman analysis, trending accuracy was based on Pearson’s moment, linear regression, and direction of change concordance. The most recent version of each device was studied. Data were collected following volume challenge and dose changes in vasoactive and inotrope therapy. As one can imagine, a lot of final data were generated from the cross comparisons. The overall conclusions were that all five methods provided tight linear correlations to changes in CO and their use could be recommended clinically. Algorithm changes had improved the performances of the PiCCOplus and FloTrac compared to previous versions. Limitations of the study were small sample size and short collection period (i.e. 2-h).

Peyton and Kozub [15] described and evaluated a prototype continuous CO method based on measuring carbon dioxide levels during ventilation where changes were induced by altering respiratory rate. Previous attempts to use a modified Fick method were performed with the NICO, now the Phillips NM3 monitor (both Philips-Respironics, Murrysville, PA, USA), which used a rebreathing loop. The new system benefits from not requiring any additional breathing circuitry or monitoring, making it easy to apply in the clinical setting. The authors provide a comprehensive description of the prototype which is a second generation version with changes to the algorithm. The prototype was tested against pulmonary artery catheter thermodilution in 52 cardiac surgery or liver transplant patients. Accepted statistical methods based on Bland–Altman, four-quadrant and polar plot analyses were used to assess agreement between methods and trending ability. The results showed that the prototype performed favorably compared to other existing CO technologies.

In one of two articles that investigated the effect of obesity on CO measurements, Altamirano-Diaz et al. [16] compared electrical cardiometry with transthoracic echocardiogram (TTE) Doppler measurements in 139 children and adolescents. They used the ICON monitor (Cardiotronic/Osypka Medical, La Jolla, CA, USA), which uses a hybrid bioimpedance method. Reasonable agreement between the cardiometry and TTE methods was shown in Bland–Altman analysis and percentage error (PE), but trending ability was not assessed. Patients were subdivided into normal weight (n = 41) and overweight/obese (n = 90). Other than a slightly greater spread of bias (agreement measures), there was little evidence that supported the author’s conclusion that obesity significantly affected ICON readings in children.

Boly et al. [17] studied the effects of morbid obesity in adults (i.e. body mass index > 35 kg/m^2^) on CO measurements using the Nexfin, which is a non-invasive pulse contour analysis method based on volume clamp finger cuff technology. Transpulmonary thermodilution was used as reference method. Data from 30 morbidly obese patients undergoing gastric bypass surgery from a previous study were re-analyzed by correcting for ideal body weight. What these authors showed was that in morbidly obese patients the Nexfin overestimates CO by up to 0.4 L/min (i.e. bias in Bland–Altman plot) and by correcting for excessive body fat agreement between measurements could be improved. The Nexfin uses the model flow algorithm that requires input of biometric data including height and weight, and adjustment of these variables can improve its reliability.

Wagner et al. [18] provided a second article that evaluated non-invasive pulse contour analysis CO. They used the CNAP (CNSystems Medizintechnik GmbH, Graz, Austria). There were two subgroups; (i) auto-calibration by inputting biometric patient data and (ii) external calibration using the first thermodilution reading from a pulmonary artery catheter, the reference method in the study. Data were collected from 51 post cardiac surgery patients. Statistical analyses were performed using Bland–Altman, PE and four-quadrant plot concordance analysis. Passive leg raise test was used to facilitate trending analysis. Not surprisingly, their data showed that the Bland–Altman agreement between methods was significantly better when coupled to the first thermodilution reading (PE = 19%) compared to the biometric calibration (PE = 49%). As well as highlighting the lack of accuracy when using patient data to calibrate this type of CO device, the study also showed that when the CNAP was calibrated to the reference method, its readings were very stable over time with little drift (i.e. PE = 19%), which indicated good trending ability. Furthermore, the concordance was 100% for both the biometric and calibrated subgroups.

In a somewhat different in-vitro study, McKenzie et al. [19] looked at the effect of different operators on the reliability of thermodilution CO measurements. Fifteen operators with varying levels of experience were recruited and parameters including hand grip strength were recorded. A mock circulation loop set at four different flow rates from 3 to 7 L/min was used to test operator performance. The authors found that experience with the technique, body mass index, and grip strength were factors that could adversely affect the accuracy of readings between operators, with a tendency to overestimate CO.

In another different type of study, Persona et al. [20] assessed whether the MostCare pulse contour analysis monitor (Vytech, Padova, Italy) could be integrated into a high-fidelity patient simulation trainer. They used the latest METI (version 6) Human Patient Simulator (Medical Education Technologies, Sarasota, FL, USA) and generated six different critical care scenarios; baseline, ventricular failure, vasoplegic shock, hypertensive crisis, hypovolemic shock, and aortic stenosis. Simulated CO hemodynamics ranged from 3 to 6.5 L/min. The MostCare could be directly connected to the METI arterial wave output and incorporated into clinical scenarios, whereas other currently available pulse contour analysis systems required dedicated catheter and transducer systems prohibiting such use. Their data showed reasonable agreement with METI COs with a PE of 19%.

## Monitoring of fluid responsiveness

The JCMC also published several papers addressing different, but highly important, aspects of fluid responsiveness prediction in 2018.

Dynamic fluid responsiveness variables, such as pulse pressure variation (PPV) and stroke volume variation (SVV), play a key role in goal-directed therapy algorithms in the perioperative setting. Since laparoscopic procedures are more and more frequently performed and since severe intra-abdominal hypertension (such as abdominal compartment syndrome) reduces the reliability of PPV and SVV, Zlicar and colleagues [21] set out to investigate the influence of pneumoperitoneum on SVV’s and PPV’s predictive values, because of the moderately increased abdominal pressure. In the analysis of 56 patients, the authors found that SVV was a reasonably reliable fluid responsiveness predictor (AUC_ROC_: 0.80), but PPV less so (AUC_ROC_: 0.67), which was in accordance with the limited data published in the field [22, 23]. The optimal SVV threshold was 12.5% with a grey zone of 7.5–13%. The applied tidal volume was 10 mL/kg probably exceeding most institutions’ guidelines and therefore the SVV threshold may need “re-calibration” in other institutions, but the study is worth reading as it elucidates important aspects of the influence of laparoscopic surgery on SVV and PPV.

Another important study was conducted by Giraud and colleagues [24]. These authors rightfully argued that the variability of superior vena cava diameter is impossible to assess with TTE, but that the variability of the subclavian vein (SCV)—given its close proximity of the superior vena cava and the pleural space—could be another way to look at these variations using ultrasound. Indeed, the authors identified an excellent ability to predict fluid responsiveness with the SCV variability in 20 ICU patients, with an optimal threshold of 14.3% (AUC_ROC_: 0.97). While close to the pleura, the SCV is located just outside the pleural space and, during controlled mechanical ventilation, the diameter of the vessel is maximal during inspiration as opposed to superior vena cava. In that sense, SCV diameter variability measurements behaves physiologically similar to inferior vena cava diameter variability, at least in terms of the respiratory phase. So, this is a quite compelling technique, not only worth a read, but also further investigation and validation.

Morparia and colleagues [25] also investigated the ability of a dynamic variable to predict fluid responsiveness. Their study was performed in a pediatric population (n = 22), which is quite important since we (as opposed to the adult population) do not have reliable fluid responsiveness prediction techniques for children. The variation in peak aortic velocity predicted fluid responsiveness very well (AUC_ROC_: 0.90) during neurosurgery, whereas PPV did not even correlate with the change in stroke volume after fluid infusion—a finding which is in line with the findings for PPV in the pediatric population. It appeared counterintuitive that the variation in peak aortic velocity in non-responders increased after fluid infusion to the baseline level of responders, underlining that this method also needs further validation. Nonetheless, it is a compelling method that should be investigated further to possibly validate a fluid responsiveness method for the pediatric (surgical) population, because it is possible that flow variations rather than pressure variations are more reliable as discussed by the authors of this worth-reading paper.

The JCMC also published a paper from Høiseth and Hagemo [26] of more theoretical character. The authors simulated in a simple manner the influence of investigating populations with either a narrow or a wide range of SVV. The authors not only concluded that a wide range of SVV values would result in better classification (as assessed by AUC_ROC_s), but also showed that the grey zone (i.e. the zone for inconclusive predictive ability) would be narrower in patients with a wide SVV range. The paper therefore highlights the important aspect of *spectrum bias*: That studies including a large portion of “extreme” values, i.e. patients where clinicians may rarely be uncertain about the fluid responsiveness state (say, initial phases of hypovolemic or distributive shock), would tend to achieve better prediction characteristics compared with studies including less extreme patients. The simulation might have been more realistic, if SVV had been simulated from a bell-shaped and right skewed distribution rather than a uniform, as evident from the reviewed papers above [24, 25], but the conclusions that classification can be influenced by spectrum bias would hardly change with such a simulation approach. This is an important aspect when assessing individual studies as well as when comparing results across studies because evaluation of spectrum bias should accompany the interpretation of AUC_ROC_s and grey zones.

## Cardiovascular physiology

A post-hoc analysis study published in the February issue by Vos et al. [27] described the importance of evaluation of the true driving force of blood circulation (the pressure gradient for venous return (Pvr), i.e. the pressure difference between Pmsa (a modified mean systemic filling pressure) and the right atrial pressure) [28], to elucidate the pathophysiological pattern associated to fluid challenge response. The authors studied 30 patients undergoing major hepatic surgery who received 15 mL/kg of fluid bolus directly after completion of hepatic resection [29]. By the way, the authors compared their finding to dynamic preload variables, such as PPV and SVV, respectively. 18/30 patients demonstrated an increase in cardiac index higher than 20% and were classified as fluid responders. There were no significant differences between all observed AUC_ROC_s. The AUC_ROC_ of Pvr in predicting fluid responsiveness was 0.75 [95% Confidence Interval (CI) 0.57–0.93; p < 0.05], the AUC_ROC_ of PPV was 0.73 [CI 0.54–0.92; p < 0.05], while that of SVV was 0.72 [CI 0.53–0.91; p < 0.05]. With their finding, the authors demonstrated that Pmsa increased in both groups following fluid administration. Yet, in responders, central venous pressure (CVP) did not change and as such, Pvr (Pmsa–CVP) increased which led to an increase in cardiac index. The finding was physiologically rational as the heart was able to generate more output from the increase in venous return [30]. In non-responders, CVP increased to a similar extent as Pmsa and the increase in CVP hinders venous return. Even if the authors demonstrated that Pvr predicts fluid responsiveness similarly to PVV and SVV, it’s important to mention: first that the setting of liver disorders and liver surgery (under general anesthesia) is a special hemodynamic condition where hypothermia [31] and cardiac dysfunction [32] impact cardiovascular coupling making dynamic indices, based on cardiopulmonary interaction, less accurate. Second that the definition of responders was based on an increase of cardiac index higher than 20%, a condition that discriminates very hypovolemic patients and by the way making Pvr indicator resolution better.

In the same February issue, Vallée et al. [33] published a pilot study in 20 patients under general anesthesia. The authors hypothesized that abdominal aortic pressure coupled with flow waveform into a pressure-flow velocity (PU) loop diagram is able to assess beat-to-beat cardiac afterload. The authors used an abdominal pressure signal recorded in the abdominal aorta coupled to a recorded flow velocity at the level of the thoracic aorta. Signals were processed through a Philips MP60 monitor (Philips, Amsterdam, The Netherlands) and a CombiQ monitor (Deltex Medical, Chichester, UK). A trans-esophageal Doppler probe (Deltex Medical, Chichester, UK) was used to assess flow velocity with the CombiQ monitor allowing to record simultaneously and continuously arterial BP and aortic velocity signals at a sampling frequency of 180 Hz. To check their hypothesis, the authors compared the PU loop recording with variables obtained from central pressure analysis estimated by non-invasive arterial tonometry. This latter non-invasive technique consisted in arterial BP signal assessed using radial applanation tonometry (SphygmoCor, AtCor Medical, Sydney, Australia), to estimate measures of cardiac afterload such as central pulse pressure and augmentation index which represents the excess pressure due to the reflected waves. The investigation was performed in both high- and low-cardiovascular risk patients during general anesthesia as well as before and after vasopressor administration. The authors demonstrated that angles derived from a PU loop are able to adequately define cardiac afterload. The results of the present work are mainly based on estimated central waveforms from transformed radial applanation tonometry, which isn’t a real gold standard [6] to derive an aortic central pressure curve.

The same team published in the October issue a study on PU loop diagrams and curves [34]. In this second study the question was if the location of pressure measurement in the PU curve is of importance and if it really affects angle values and markers derived from PU loops. The authors studied 25 patients (with high- or low-cardiovascular risks) undergoing elective or emergency neuroradiology interventions under general anesthesia. The originality of the study was that they had the possibility to measure invasive thoracic aortic pressure, invasive radial arterial BP, and reconstructed aortic pressure obtained by applying a transfer function on peripheral radial pressure. The results of the study show there are differences in angle values if the PU loop is built using a radial pressure waveform as compared with using a pressure waveform measured in the descending thoracic aorta (central pressure). Moreover, construction of PU loops from radial artery pressure resulted in an underestimation of both angles and this underestimation was greater in patients with higher cardiovascular risks. However, interestingly, this underestimation seems to be corrected with a transfer function estimation of aortic pressures from the radial catheter signal. In their discussion the authors acknowledged the main limitation of the investigation, the use of a fluid filled catheter as opposed to micro-manometer sensors to measure central arterial pressure. The other limitation was the absence of measurements and analysis during therapeutic interventions like a change in arterial tone or venous return [35].

## Technical developments

Through 2018, we also saw a few papers in the area of technical development. Fujiwara and colleagues [36] validated a newly developed three-way stopcock and showed that the natural frequency and damping coefficients were not altered to a clinically relevant extent even when three stopcocks were inserted serially before the pressure transducer. The new stopcock appeared a safe substitute for existing stop-cocks in terms of the frequency response, while also offering improved features to help preventing contamination.

Another important study published in 2018 was Xu and colleagues’ description [37] of a novel device for air removal from infusion lines. At high infusion rates (250–750 mL/min), the new device removed air in all the investigated in vitro setups, where air was artificially added during the simulated infusions, and therefore resulted in no infusion pauses. This contrasted the comparative infusion pump, where air bubbles were not efficiently removed and therefore detected by the air detector resulting in 60% “pause-time” of the infusion pump. In highly acute settings, e.g. severe trauma, such a new devise may play an important role in avoiding pause-time of high-flow infusion pumps, and the device was therefore patented by the authors.

In terms of novel algorithms, Rebergen and colleagues [38] published the methodology of a simple algorithm for the detection of artifacts in the R-R interval time series, which is highly relevant for the quantification of heart rate variability. The authors correctly assumed that very fast fluctuations in the R-R interval time series (absolute differences) would identify both missed R spikes as well as erroneous “premature” detections and showed that this simple approach, which is patient-independent, outperformed two existing algorithms currently used and described in the scientific literature. The algorithm was developed solely from electrocardiograms (ECG) of subarachnoid hemorrhage patients admitted to a neurological ICU—for which the methodology is intended—and the algorithm worked almost perfectly in this setting, but may not be applicable in other (e.g. healthy) populations without a re-calibration of the identified thresholds.

## Closed loop hemodynamic management

Three articles from the 2018 volume of the JCMC deal with the issue of closed-loop hemodynamic management. Automation is everywhere and is currently also being introduced in medicine for better and more consistent patient care while simultaneously reducing workload and potential for errors. In the February issue, Rinehart and coworkers [39] presented an in-silico study of 250 simulated cases where they aimed to maintain a target BP by using a closed-loop processor-controlled vasopressor infusion both in hemodynamically stable and variable BP conditions. The vasopressor titration controller, developed by the group of authors, received BP values as input, got a target mean arterial BP of 70 mmHg with a tolerance zone of ± 5 mmHg, and produced a vasopressor infusion rate as output. This information was fed into a patient state simulator, which “responded” to the vasopressor dose and sent the new vital signs back to the controller, which in turn adjusts its vasopressor infusion rate, closing the loop. The controller was tested in a simulated physiological model at two scenarios, one with a stable and one with a variable mean BP. The controller was able to keep the mean BP (± SD) at 70.3 ± 2.6 mmHg in the stable conditions and at 70.5 ± 3.2 mmHg in the variable conditions. The time the simulated cases spent in the target pressure range were 99.5% and 88.6% in the stable and unstable hemodynamic conditions, respectively. Low BPs were corrected faster than high values, probably because vasopressor infusion was the simulated intervention. These results show that maintaining BP within predefined targets via a vasopressor titration controller is feasible, even if mean BP fluctuated at random in clinically relevant magnitudes, simulating hemodynamic instability. The authors did not hesitate to mention that this elegant piece of automating BP control cannot be seen as a definite solution to all kinds of clinical hypotensive events, such as caused by hypovolemia or cardiac pump failure, and that therefore clinical judgment, diagnosis and decision making to intervene is still required.

Closed-loop controllers can naturally also be used for the reverse purpose, i.e. the prevention of hypertension, which usually occurs after brain death during catecholamine storm. In this case, the controller regulates an infusion pump with a vasodilator. This approach was chosen by Soltesz et al. [40] who used an experimental model of brain dead pigs. Their closed-loop hemodynamic stabilization system consisted of two loops, one with a norepinephrine pump to avoid hypotension, and one with a nitroglycerine pump to treat hypertension. They reported that the increase in arterial BP associated with the catecholamine storm was almost completely eliminated by the controller, resulting in a mean BP being within the predefined target range for 98% of the time. The norepinephrine pump did not have to be activated, since no hypotension occurred in this setting.

The final step, after in silico and animal experiments, is testing automated closed-loop controlling systems in patients.

In the December issue of the journal, Lilot et al. [41] published a clinical case-control study in which they compared CO values of 46 patients undergoing abdominal surgery randomized to be managed with a closed-loop system for goal-directed fluid therapy (GDFT) with CO of patients treated with usual care without a GDFT protocol in their academic center. The system relies on an algorithm using SVV as a trigger to automatically apply fluids via a pump to reach a preselected CO target. The authors hypothesized that their closed-loop system would provide a better CO optimization as compared with usual practice. The closed-loop system administered on average 8.5 (range 0 to 34) fluid boluses of 100 mL colloid to each patient in the closed-loop GDFT group. The system was overruled by the attending anesthesiologists in 12 instances, 8 times to force an additional fluid bolus administration and four times to halt a bolus planned by the system. Yet, there were no difference in intraoperative CO between groups, although initial CO was lower in the closed-loop group, indicating that this group was more likely hypovolemic. Nevertheless, both groups received roughly the same amounts of volume intraoperatively. Furthermore, both groups spent most of the intraoperative period in a fluid unresponsive state (91% and 83% for the closed-loop and control group, respectively). Although this study failed to show superior hemodynamics in the closed-loop GDFT group due to an unexpected lower initial CO in this group, it confirms that such systems could be safely used to help the care provider making decisions on fluid optimization to improve hemodynamics.

## Perioperative outcome

Two author groups were investigating the effects of hemodynamic monitoring on perioperative patient outcome. In the first paper, Leclercq et al. [42] present an observational case-control study in which they investigated the feasibility and clinical utility of the endotracheal CO monitor (ECOM), a bioimpedance cardiography based method incorporated in an endotracheal tube, to optimize intraoperative hemodynamics and improve short-term outcome in 20 patients undergoing elective off-pump coronary artery bypass grafting (OPCAB). For this purpose, patients were equipped with a special commercially available endotracheal tube containing seven electrodes measuring the bioimpedance signal from the ascending aorta, which is in close proximity to the trachea. The authors studied outcomes such as postoperative admission to the ICU (as assessed by an independent physician blinded to the study endpoints), time to extubation and length of stay in ICU and hospital. The results were compared to a retrospective control group of patients undergoing the same type of operation without ECOM monitoring. While 90% of the control patients were admitted to the ICU, only 11/20 (55%) patients from the ECOM group required ICU admission. This difference was mainly caused by an unexpected rate of ICU admission related to hemodynamic instability in the control group, with zero admissions due to that reason in the monitored group. Also, the time to extubation, the length of stay in the ICU, and both troponin and lactate levels were all significantly decreased in the ECOM group. The authors concluded that the use of ECOM monitoring was associated with an improvement in short-term outcome after OPCAB, although the sample size may be too small to draw such firm conclusions. As the pure use of a monitoring device per se cannot affect outcome, the effects are probably related to the therapeutic consequences based on its measurements, such as fluid management and vasoactive medications, since the doctors involved in that study were familiar with goal-directed therapy algorithms, although no strict protocol was used. Finally, the results of this study should be confirmed in a setting where transesophageal echocardiography is established in the hemodynamic management of OPCAB patients.

The second outcome-related article is a state-of-the-art systematic review and meta-analysis on the effects of early goal-directed hemodynamic therapy (EGDT) on all-cause mortality in critically ill patients [43]. The authors screened 998 papers from the literature, of which 13 met the inclusion criteria for the systematic review and meta-analysis. These studies included a total of 6850 patients. The authors further divided the trials in those having a low or high risk of bias based on the Cochrane collaborative tool for risk of bias assessment. Six trials including 3323 patients were classified as having low risk of bias, and there was no difference in mortality between the intervention and the control group (22.4% vs. 22.9%, odds ratio 0.94 with 95% CI 0.73–1.22). Similar results were found when all 13 studies were analyzed (mortality of 23.8% vs. 23.5% in intervention and control group, respectively, odds ratio 1.0 with 95% CI 0.89–1.12), and when studies using less invasive monitoring techniques such as bioreactance and trans-esophageal Doppler were excluded (mortality of 24.8% vs. 24.3% in intervention and control group, respectively, odds ratio 1.01 with 95% CI 0.89–1.13). The authors also performed a trial sequential analysis, which revealed that a total of 17,532 patients would have been needed to be able to exclude that the negative findings were random. The quality of evidence as assessed using the Grades of Recommendation Assessment, Development and Evaluation system was considered as moderate. The authors concluded that the sample size of the meta-analysis was too small to exclude that the negative effects found were by chance, and that the relatively low mortality in the control groups might have attenuated the effects of the interventions. This was certainly not the first systematic review and meta-analysis on the effects of EGDT on mortality in critically ill patients and previous meta-analysis revealed conflicting results, i.e. either a superiority [44] or non-superiority of EGDT [45, 46]. Of note, a recent systematic review from the perioperative setting refrained from pooling the data of the single studies to perform a meta-analysis due to significant heterogeneity in patients, interventions and outcomes between the different studies [47].

## Intraoperative hypotension

Induction and maintenance of general anesthesia is often associated with a decrease in BP. Several studies provide evidence that hypotension is associated with postoperative myocardial injury [48], acute kidney injury [49–51], and death [52, 53]. We would like to highlight some articles which focused on hypotension during the perioperative period.

In the June issue, Juri et al. [54] presented a study with 45 patients undergoing abdominal surgery. The authors investigated the reliability of pre-anesthetic SVV and pleth variability index (PVI) for the prediction of hypotension and a decrease in CO induced by the induction of general anesthesia. SVV was assessed by electrical cardiometry, PVI using pulse oximetry. The patients were subsequently divided into a high SVV group (pre-anesthetic SVV > 12%) and a low SVV group (pre-anesthetic SVV ≤ 12%). The study showed that in the high SVV group more patients had a decrease in CO compared to the low SVV group (92.0% vs. 40.0%) following induction of general anesthesia, defined as a reduction to less than 70% of baseline CO. Additionally, the minimum CO (± SD) during the procedure was lower in patients with high pre-anesthetic SVV compared to patients in the low SVV group (2.70 ± 0.70 L/min vs. 3.16 ± 0.63 L/min, p < 0.05) indicating a greater CO decrease (39.4% ± 7.7% vs. 30.1% ± 12.4%, p < 0.05) as baseline CO did not differ between groups. Receiver operating curves revealed that in this cohort a SVV > 13% predicted a CO decrease > 30% during anesthesia induction with a sensitivity of 83.9% and specificity of 78.6%. A multivariate logistic regression analysis revealed that high pre-anesthetic SVV was an independent prognostic factor for a decrease in CO and BP during induction of general anesthesia. The authors concluded that pre-induction assessment of SVV can help to induce prophylactic actions like volume expansion or vasopressor administration to avoid decreased CO and hypotension during anesthesia induction.

Another study with focus on hypotension after induction of anesthesia was performed by Padley et al. [55] who obtained an ECG in 31 patients before major abdominal surgery. ECGs were recorded a median of three days before surgery to derive heart rate variability parameters for comparison of patients who experienced post-induction hypotension with patients maintaining stable hemodynamics. The analyses included time domain and frequency-domain as well as non-linear heart rate variability indices. The data showed that post-induction hypotension occurred relatively frequently (45%). These hypotensive patients had lower pre-operative heart rate variability and spectral power, a higher low frequency/high frequency-ratio, and reduced heart rate complexity as defined by the correlation dimension. Also, a higher American Society of Anesthesiologists (ASA) classification was found to be a predictor of hypotension in this study, as previously described [56]. Limitations of this study were the relatively small sample size and the exclusion of six patients with failed heart rate variability measurements due to artifacts or ectopy. Nonetheless, the data indicate that ECG could serve as a useful screening tool for post-induction hypotension in patients undergoing major abdominal surgery. Further trials are warranted to test, whether these results can be confirmed in larger patient populations.

In order to treat or even prevent hypotension after induction of general anesthesia, vasopressors are often administered to increase hemodynamic stability. Phenylephrine is known to increase systemic vascular resistance and arterial BP, however, the effects on other hemodynamic variables still need to be elucidated. Kalmar et al. [57] performed a prospective trial to investigate phenylephrine, a direct α-agonist. The authors included 18 patients scheduled for elective laparoscopic sigmoidectomy. After the placement of an epidural catheter and induction of general anesthesia, patients were equipped with an arterial catheter for continuous BP measurement. The ProAQT transducer of the PulsioFlex monitor (Pulsion Medical Systems SE) was used to calculate additional hemodynamic variables, e.g. stroke volume and CO. To determine cardiac preload, the dynamic cardiac preload variables PPV and SVV were derived as well as systemic vascular resistance for left ventricular afterload. If mean arterial BP dropped below 80% of the awake baseline value, a bolus of 2 µg/kg-bodyweight phenylephrine was administered. Mean (± SD) CO increased from 3.92 ± 0.87 L/min to 4.94 ± 1.20 L/min after the phenylephrine administration. Mean arterial BP, stroke volume, CVP, and etCO_2_ also increased, while PPV and SVV decreased. The authors concluded that in patients with preload dependency, defined as PPV > 12%, and anesthesia-induced hypotension, phenylephrine increases cardiac filling and subsequently CO. Even though no echocardiographic measurements were performed to detect right and left ventricular volumes, the study creates better understanding of the effects of phenylephrine. In the discussion, the authors underline the importance of advanced hemodynamic monitoring to prevent organ damage and guidance of hemodynamic management.

In patients under general or regional anesthesia the interval for non-invasive BP measurements is recommended to be not longer than 5 min to monitor hemodynamic function. However, prolonged intervals between BP measurements due to technical issues or other factors may occur and increase the risk of undetected hypotension or hemodynamic instability. To investigate whether prolonged BP measurement intervals and hypotension are related, Kruger et al. [58] performed a retrospective database analysis including 139,509 adult patients having surgery under general anesthesia. Additionally, the data from the anesthesia information management system were analyzed using a logistic regression model to identify predictors of prolonged intervals between BP measurements. Over 5 million BP measurements were included in the analysis that revealed that 0.8% of the measured intervals were > 6 min and only 0.2% were > 10 min. These prolonged measurement intervals were associated with an increased risk of hypotension, however the authors point out that prolonged measurement intervals are not causing hypotension. Logistic regression models showed that age, higher ASA classification, obesity, and repositioning of the patient were identified as predictors for prolonged BP measurement intervals. The study shows that prolonged BP measurement intervals have not disappeared with technical advancement. Nevertheless, modern technical solutions allow more frequent BP measurements and alerting strategies for prolonged intervals may help to reduce the incidence of hypotension.
